# Supplementary material for: Pyruvate Dehydrogenase Kinase 1 inhibition mediated oxidative phosphorylation enhancement in cartilage promotes osteoarthritis progression
Source: BMC Musculoskelet Disord. 2023 Jul 20;24:597. doi: 10.1186/s12891-023-06585-6 (PMC10357736; doi:10.1186/s12891-023-06585-6)
Supplement: Supplementary file 1 — Additional file 1. [file 12891_2023_6585_MOESM1_ESM.zip › MF.Gsea.1653627316242/GOMF_HEXOSYLTRANSFERASE_ACTIVITY.html]

Details for gene set GOMF\_HEXOSYLTRANSFERASE\_ACTIVITY[GSEA]

|  || Dataset | 55457\_collapsed\_to\_symbols.cls.cls #OA\_versus\_normal.cls.cls #OA\_versus\_normal\_repos |
| Phenotype | cls.cls#OA\_versus\_normal\_repos |
| Upregulated in class | OA |
| GeneSet | GOMF\_HEXOSYLTRANSFERASE\_ACTIVITY |
| Enrichment Score (ES) | 0.2865319 |
| Normalized Enrichment Score (NES) | 1.4041125 |
| Nominal p-value | 0.02601156 |
| FDR q-value | 0.3214055 |
| FWER p-Value | 1.0 |
Table: GSEA Results Summary

  

Fig 1: Enrichment plot: GOMF\_HEXOSYLTRANSFERASE\_ACTIVITY      
 Profile of the Running ES Score & Positions of GeneSet Members on the Rank Ordered List

  

| SYMBOL | TITLE | RANK IN GENE LIST | RANK METRIC SCORE | RUNNING ES | CORE ENRICHMENT || 1 | C1GALT1C1 | C1GALT1 specific chaperone 1 [Source:HGNC Symbol;Acc:HGNC:24338] | 22 | 1.098 | 0.0244 | Yes |
| 2 | B3GALNT1 | "beta-1,3-N-acetylgalactosaminyltransferase 1 (globoside blood group) [Source:HGNC Symbol;Acc:HGNC:918]" | 101 | 0.914 | 0.0401 | Yes |
| 3 | GALNT11 | polypeptide N-acetylgalactosaminyltransferase 11 [Source:HGNC Symbol;Acc:HGNC:19875] | 164 | 0.845 | 0.0553 | Yes |
| 4 | EXT2 | exostosin glycosyltransferase 2 [Source:HGNC Symbol;Acc:HGNC:3513] | 170 | 0.838 | 0.0749 | Yes |
| 5 | MGAT4C | MGAT4 family member C [Source:HGNC Symbol;Acc:HGNC:30871] | 234 | 0.796 | 0.0889 | Yes |
| 6 | B4GAT1 | "beta-1,4-glucuronyltransferase 1 [Source:HGNC Symbol;Acc:HGNC:15685]" | 282 | 0.764 | 0.1034 | Yes |
| 7 | EXTL2 | exostosin like glycosyltransferase 2 [Source:HGNC Symbol;Acc:HGNC:3516] | 357 | 0.722 | 0.1148 | Yes |
| 8 | PIGP | phosphatidylinositol glycan anchor biosynthesis class P [Source:HGNC Symbol;Acc:HGNC:3046] | 367 | 0.718 | 0.1312 | Yes |
| 9 | UGT2B4 | UDP glucuronosyltransferase family 2 member B4 [Source:HGNC Symbol;Acc:HGNC:12553] | 566 | 0.634 | 0.1308 | Yes |
| 10 | MGAT4A | "alpha-1,3-mannosyl-glycoprotein 4-beta-N-acetylglucosaminyltransferase A [Source:HGNC Symbol;Acc:HGNC:7047]" | 657 | 0.604 | 0.1381 | Yes |
| 11 | ALG6 | "ALG6 alpha-1,3-glucosyltransferase [Source:HGNC Symbol;Acc:HGNC:23157]" | 696 | 0.595 | 0.1493 | Yes |
| 12 | ALG8 | "ALG8 alpha-1,3-glucosyltransferase [Source:HGNC Symbol;Acc:HGNC:23161]" | 900 | 0.543 | 0.1463 | Yes |
| 13 | ALG5 | ALG5 dolichyl-phosphate beta-glucosyltransferase [Source:HGNC Symbol;Acc:HGNC:20266] | 945 | 0.529 | 0.1555 | Yes |
| 14 | B3GALT4 | "beta-1,3-galactosyltransferase 4 [Source:HGNC Symbol;Acc:HGNC:919]" | 962 | 0.526 | 0.1668 | Yes |
| 15 | HYAL1 | hyaluronidase 1 [Source:HGNC Symbol;Acc:HGNC:5320] | 997 | 0.518 | 0.1764 | Yes |
| 16 | PIGV | phosphatidylinositol glycan anchor biosynthesis class V [Source:HGNC Symbol;Acc:HGNC:26031] | 1064 | 0.503 | 0.1832 | Yes |
| 17 | C1GALT1 | "core 1 synthase, glycoprotein-N-acetylgalactosamine 3-beta-galactosyltransferase 1 [Source:HGNC Symbol;Acc:HGNC:24337]" | 1110 | 0.494 | 0.1915 | Yes |
| 18 | GALNT10 | polypeptide N-acetylgalactosaminyltransferase 10 [Source:HGNC Symbol;Acc:HGNC:19873] | 1173 | 0.481 | 0.1981 | Yes |
| 19 | GYG1 | glycogenin 1 [Source:HGNC Symbol;Acc:HGNC:4699] | 1209 | 0.474 | 0.2066 | Yes |
| 20 | RPN2 | ribophorin II [Source:HGNC Symbol;Acc:HGNC:10382] | 1294 | 0.459 | 0.2110 | Yes |
| 21 | B4GALT7 | "beta-1,4-galactosyltransferase 7 [Source:HGNC Symbol;Acc:HGNC:930]" | 1411 | 0.442 | 0.2124 | Yes |
| 22 | B3GNT2 | "UDP-GlcNAc:betaGal beta-1,3-N-acetylglucosaminyltransferase 2 [Source:HGNC Symbol;Acc:HGNC:15629]" | 1427 | 0.439 | 0.2217 | Yes |
| 23 | HEXB | hexosaminidase subunit beta [Source:HGNC Symbol;Acc:HGNC:4879] | 1434 | 0.438 | 0.2316 | Yes |
| 24 | ALG9 | "ALG9 alpha-1,2-mannosyltransferase [Source:HGNC Symbol;Acc:HGNC:15672]" | 1487 | 0.431 | 0.2378 | Yes |
| 25 | ABO | "ABO, alpha 1-3-N-acetylgalactosaminyltransferase and alpha 1-3-galactosyltransferase [Source:HGNC Symbol;Acc:HGNC:79]" | 1495 | 0.429 | 0.2475 | Yes |
| 26 | B4GALT6 | "beta-1,4-galactosyltransferase 6 [Source:HGNC Symbol;Acc:HGNC:929]" | 1500 | 0.429 | 0.2574 | Yes |
| 27 | FUT8 | fucosyltransferase 8 [Source:HGNC Symbol;Acc:HGNC:4019] | 1565 | 0.418 | 0.2623 | Yes |
| 28 | GCNT3 | "glucosaminyl (N-acetyl) transferase 3, mucin type [Source:HGNC Symbol;Acc:HGNC:4205]" | 1683 | 0.401 | 0.2627 | Yes |
| 29 | PYGM | "glycogen phosphorylase, muscle associated [Source:HGNC Symbol;Acc:HGNC:9726]" | 1952 | 0.367 | 0.2505 | Yes |
| 30 | GCNT1 | glucosaminyl (N-acetyl) transferase 1 [Source:HGNC Symbol;Acc:HGNC:4203] | 2035 | 0.357 | 0.2526 | Yes |
| 31 | UGT2B17 | UDP glucuronosyltransferase family 2 member B17 [Source:HGNC Symbol;Acc:HGNC:12547] | 2050 | 0.356 | 0.2599 | Yes |
| 32 | CHPF2 | chondroitin polymerizing factor 2 [Source:HGNC Symbol;Acc:HGNC:29270] | 2281 | 0.329 | 0.2498 | Yes |
| 33 | FUT4 | fucosyltransferase 4 [Source:HGNC Symbol;Acc:HGNC:4015] | 2318 | 0.324 | 0.2547 | Yes |
| 34 | FUT7 | fucosyltransferase 7 [Source:HGNC Symbol;Acc:HGNC:4018] | 2343 | 0.322 | 0.2604 | Yes |
| 35 | PYGB | glycogen phosphorylase B [Source:HGNC Symbol;Acc:HGNC:9723] | 2400 | 0.315 | 0.2635 | Yes |
| 36 | CSGALNACT1 | chondroitin sulfate N-acetylgalactosaminyltransferase 1 [Source:HGNC Symbol;Acc:HGNC:24290] | 2507 | 0.302 | 0.2624 | Yes |
| 37 | PIGH | phosphatidylinositol glycan anchor biosynthesis class H [Source:HGNC Symbol;Acc:HGNC:8964] | 2573 | 0.294 | 0.2643 | Yes |
| 38 | RFNG | RFNG O-fucosylpeptide 3-beta-N-acetylglucosaminyltransferase [Source:HGNC Symbol;Acc:HGNC:9974] | 2646 | 0.286 | 0.2655 | Yes |
| 39 | PIGB | phosphatidylinositol glycan anchor biosynthesis class B [Source:HGNC Symbol;Acc:HGNC:8959] | 2663 | 0.284 | 0.2710 | Yes |
| 40 | UGT2A3 | UDP glucuronosyltransferase family 2 member A3 [Source:HGNC Symbol;Acc:HGNC:28528] | 2854 | 0.265 | 0.2624 | Yes |
| 41 | B3GALT2 | "beta-1,3-galactosyltransferase 2 [Source:HGNC Symbol;Acc:HGNC:917]" | 2883 | 0.262 | 0.2665 | Yes |
| 42 | GALNT7 | polypeptide N-acetylgalactosaminyltransferase 7 [Source:HGNC Symbol;Acc:HGNC:4129] | 2954 | 0.255 | 0.2671 | Yes |
| 43 | B3GAT3 | "beta-1,3-glucuronyltransferase 3 [Source:HGNC Symbol;Acc:HGNC:923]" | 2987 | 0.251 | 0.2705 | Yes |
| 44 | HEXA | hexosaminidase subunit alpha [Source:HGNC Symbol;Acc:HGNC:4878] | 3025 | 0.247 | 0.2735 | Yes |
| 45 | PLOD1 | "procollagen-lysine,2-oxoglutarate 5-dioxygenase 1 [Source:HGNC Symbol;Acc:HGNC:9081]" | 3155 | 0.235 | 0.2690 | Yes |
| 46 | POGLUT2 | protein O-glucosyltransferase 2 [Source:HGNC Symbol;Acc:HGNC:19350] | 3238 | 0.227 | 0.2680 | Yes |
| 47 | UGT1A8 | UDP glucuronosyltransferase family 1 member A8 [Source:HGNC Symbol;Acc:HGNC:12540] | 3301 | 0.221 | 0.2684 | Yes |
| 48 | EXT1 | exostosin glycosyltransferase 1 [Source:HGNC Symbol;Acc:HGNC:3512] | 3321 | 0.219 | 0.2721 | Yes |
| 49 | MTAP | methylthioadenosine phosphorylase [Source:HGNC Symbol;Acc:HGNC:7413] | 3324 | 0.219 | 0.2772 | Yes |
| 50 | GBA | glucosylceramidase beta [Source:HGNC Symbol;Acc:HGNC:4177] | 3327 | 0.219 | 0.2822 | Yes |
| 51 | B4GALT4 | "beta-1,4-galactosyltransferase 4 [Source:HGNC Symbol;Acc:HGNC:927]" | 3407 | 0.211 | 0.2810 | Yes |
| 52 | MFNG | MFNG O-fucosylpeptide 3-beta-N-acetylglucosaminyltransferase [Source:HGNC Symbol;Acc:HGNC:7038] | 3442 | 0.207 | 0.2833 | Yes |
| 53 | AGL | "amylo-alpha-1, 6-glucosidase, 4-alpha-glucanotransferase [Source:HGNC Symbol;Acc:HGNC:321]" | 3537 | 0.199 | 0.2807 | Yes |
| 54 | TYMP | thymidine phosphorylase [Source:HGNC Symbol;Acc:HGNC:3148] | 3588 | 0.195 | 0.2814 | Yes |
| 55 | PLOD3 | "procollagen-lysine,2-oxoglutarate 5-dioxygenase 3 [Source:HGNC Symbol;Acc:HGNC:9083]" | 3614 | 0.192 | 0.2840 | Yes |
| 56 | POMGNT1 | "protein O-linked mannose N-acetylglucosaminyltransferase 1 (beta 1,2-) [Source:HGNC Symbol;Acc:HGNC:19139]" | 3641 | 0.191 | 0.2865 | Yes |
| 57 | ALG12 | "ALG12 alpha-1,6-mannosyltransferase [Source:HGNC Symbol;Acc:HGNC:19358]" | 3723 | 0.183 | 0.2846 | No |
| 58 | PYGL | glycogen phosphorylase L [Source:HGNC Symbol;Acc:HGNC:9725] | 3826 | 0.174 | 0.2807 | No |
| 59 | GCNT4 | glucosaminyl (N-acetyl) transferase 4 [Source:HGNC Symbol;Acc:HGNC:17973] | 3858 | 0.169 | 0.2823 | No |
| 60 | DPY19L1 | dpy-19 like C-mannosyltransferase 1 [Source:HGNC Symbol;Acc:HGNC:22205] | 3875 | 0.167 | 0.2850 | No |
| 61 | ALG3 | "ALG3 alpha-1,3- mannosyltransferase [Source:HGNC Symbol;Acc:HGNC:23056]" | 4096 | 0.146 | 0.2713 | No |
| 62 | STT3A | STT3 oligosaccharyltransferase complex catalytic subunit A [Source:HGNC Symbol;Acc:HGNC:6172] | 4618 | 0.103 | 0.2329 | No |
| 63 | COLGALT2 | collagen beta(1-O)galactosyltransferase 2 [Source:HGNC Symbol;Acc:HGNC:16790] | 4703 | 0.096 | 0.2286 | No |
| 64 | B3GAT1 | "beta-1,3-glucuronyltransferase 1 [Source:HGNC Symbol;Acc:HGNC:921]" | 4715 | 0.095 | 0.2300 | No |
| 65 | PIGQ | phosphatidylinositol glycan anchor biosynthesis class Q [Source:HGNC Symbol;Acc:HGNC:14135] | 4825 | 0.085 | 0.2235 | No |
| 66 | UGT1A10 | UDP glucuronosyltransferase family 1 member A10 [Source:HGNC Symbol;Acc:HGNC:12531] | 4850 | 0.083 | 0.2236 | No |
| 67 | POFUT1 | protein O-fucosyltransferase 1 [Source:HGNC Symbol;Acc:HGNC:14988] | 4910 | 0.079 | 0.2209 | No |
| 68 | MGAT5 | "alpha-1,6-mannosylglycoprotein 6-beta-N-acetylglucosaminyltransferase [Source:HGNC Symbol;Acc:HGNC:7049]" | 5086 | 0.066 | 0.2087 | No |
| 69 | B3GALT5 | "beta-1,3-galactosyltransferase 5 [Source:HGNC Symbol;Acc:HGNC:920]" | 5132 | 0.061 | 0.2067 | No |
| 70 | DPY19L4 | dpy-19 like 4 [Source:HGNC Symbol;Acc:HGNC:27829] | 5133 | 0.061 | 0.2081 | No |
| 71 | MGAT2 | "alpha-1,6-mannosyl-glycoprotein 2-beta-N-acetylglucosaminyltransferase [Source:HGNC Symbol;Acc:HGNC:7045]" | 5317 | 0.046 | 0.1949 | No |
| 72 | MGAT1 | "alpha-1,3-mannosyl-glycoprotein 2-beta-N-acetylglucosaminyltransferase [Source:HGNC Symbol;Acc:HGNC:7044]" | 5407 | 0.040 | 0.1889 | No |
| 73 | GALNT4 | polypeptide N-acetylgalactosaminyltransferase 4 [Source:HGNC Symbol;Acc:HGNC:4126] | 5610 | 0.023 | 0.1736 | No |
| 74 | GYG2 | glycogenin 2 [Source:HGNC Symbol;Acc:HGNC:4700] | 5756 | 0.011 | 0.1625 | No |
| 75 | B3GALT1 | "beta-1,3-galactosyltransferase 1 [Source:HGNC Symbol;Acc:HGNC:916]" | 5864 | 0.004 | 0.1542 | No |
| 76 | UGT8 | UDP glycosyltransferase 8 [Source:HGNC Symbol;Acc:HGNC:12555] | 5908 | 0.002 | 0.1509 | No |
| 77 | GALNT6 | polypeptide N-acetylgalactosaminyltransferase 6 [Source:HGNC Symbol;Acc:HGNC:4128] | 5915 | 0.001 | 0.1505 | No |
| 78 | MGAT4B | "alpha-1,3-mannosyl-glycoprotein 4-beta-N-acetylglucosaminyltransferase B [Source:HGNC Symbol;Acc:HGNC:7048]" | 5956 | -0.002 | 0.1474 | No |
| 79 | LFNG | LFNG O-fucosylpeptide 3-beta-N-acetylglucosaminyltransferase [Source:HGNC Symbol;Acc:HGNC:6560] | 6161 | -0.017 | 0.1318 | No |
| 80 | EXTL1 | exostosin like glycosyltransferase 1 [Source:HGNC Symbol;Acc:HGNC:3515] | 6193 | -0.020 | 0.1299 | No |
| 81 | GALNT1 | polypeptide N-acetylgalactosaminyltransferase 1 [Source:HGNC Symbol;Acc:HGNC:4123] | 6217 | -0.022 | 0.1286 | No |
| 82 | GYS2 | glycogen synthase 2 [Source:HGNC Symbol;Acc:HGNC:4707] | 6316 | -0.029 | 0.1216 | No |
| 83 | COLGALT1 | collagen beta(1-O)galactosyltransferase 1 [Source:HGNC Symbol;Acc:HGNC:26182] | 6535 | -0.045 | 0.1056 | No |
| 84 | RPN1 | ribophorin I [Source:HGNC Symbol;Acc:HGNC:10381] | 6618 | -0.051 | 0.1004 | No |
| 85 | POMT1 | protein O-mannosyltransferase 1 [Source:HGNC Symbol;Acc:HGNC:9202] | 6652 | -0.053 | 0.0991 | No |
| 86 | GALNT14 | polypeptide N-acetylgalactosaminyltransferase 14 [Source:HGNC Symbol;Acc:HGNC:22946] | 6762 | -0.060 | 0.0920 | No |
| 87 | LALBA | lactalbumin alpha [Source:HGNC Symbol;Acc:HGNC:6480] | 7143 | -0.092 | 0.0644 | No |
| 88 | LARGE1 | LARGE xylosyl- and glucuronyltransferase 1 [Source:HGNC Symbol;Acc:HGNC:6511] | 7164 | -0.093 | 0.0650 | No |
| 89 | UGT2B11 | UDP glucuronosyltransferase family 2 member B11 [Source:HGNC Symbol;Acc:HGNC:12545] | 7270 | -0.100 | 0.0592 | No |
| 90 | GYS1 | glycogen synthase 1 [Source:HGNC Symbol;Acc:HGNC:4706] | 7345 | -0.105 | 0.0559 | No |
| 91 | GALNT2 | polypeptide N-acetylgalactosaminyltransferase 2 [Source:HGNC Symbol;Acc:HGNC:4124] | 7453 | -0.115 | 0.0503 | No |
| 92 | DPM1 | "dolichyl-phosphate mannosyltransferase subunit 1, catalytic [Source:HGNC Symbol;Acc:HGNC:3005]" | 7723 | -0.135 | 0.0324 | No |
| 93 | B4GALNT1 | "beta-1,4-N-acetyl-galactosaminyltransferase 1 [Source:HGNC Symbol;Acc:HGNC:4117]" | 7799 | -0.141 | 0.0299 | No |
| 94 | CHPF | chondroitin polymerizing factor [Source:HGNC Symbol;Acc:HGNC:24291] | 7960 | -0.152 | 0.0210 | No |
| 95 | GALNT8 | polypeptide N-acetylgalactosaminyltransferase 8 [Source:HGNC Symbol;Acc:HGNC:4130] | 8124 | -0.164 | 0.0121 | No |
| 96 | B3GNT3 | "UDP-GlcNAc:betaGal beta-1,3-N-acetylglucosaminyltransferase 3 [Source:HGNC Symbol;Acc:HGNC:13528]" | 8361 | -0.183 | -0.0020 | No |
| 97 | UGGT2 | UDP-glucose glycoprotein glucosyltransferase 2 [Source:HGNC Symbol;Acc:HGNC:15664] | 8568 | -0.199 | -0.0134 | No |
| 98 | GCNT2 | glucosaminyl (N-acetyl) transferase 2 (I blood group) [Source:HGNC Symbol;Acc:HGNC:4204] | 8619 | -0.202 | -0.0125 | No |
| 99 | FUT5 | fucosyltransferase 5 [Source:HGNC Symbol;Acc:HGNC:4016] | 8898 | -0.225 | -0.0289 | No |
| 100 | MGAT3 | "beta-1,4-mannosyl-glycoprotein 4-beta-N-acetylglucosaminyltransferase [Source:HGNC Symbol;Acc:HGNC:7046]" | 8920 | -0.226 | -0.0252 | No |
| 101 | PIGZ | phosphatidylinositol glycan anchor biosynthesis class Z [Source:HGNC Symbol;Acc:HGNC:30596] | 9047 | -0.235 | -0.0295 | No |
| 102 | B4GALT5 | "beta-1,4-galactosyltransferase 5 [Source:HGNC Symbol;Acc:HGNC:928]" | 9051 | -0.236 | -0.0241 | No |
| 103 | POMT2 | protein O-mannosyltransferase 2 [Source:HGNC Symbol;Acc:HGNC:19743] | 9216 | -0.249 | -0.0310 | No |
| 104 | FUT9 | fucosyltransferase 9 [Source:HGNC Symbol;Acc:HGNC:4020] | 9433 | -0.268 | -0.0415 | No |
| 105 | ALG13 | ALG13 UDP-N-acetylglucosaminyltransferase subunit [Source:HGNC Symbol;Acc:HGNC:30881] | 9444 | -0.268 | -0.0359 | No |
| 106 | UGT2A1 | UDP glucuronosyltransferase family 2 member A1 complex locus [Source:HGNC Symbol;Acc:HGNC:12542] | 9451 | -0.269 | -0.0300 | No |
| 107 | B3GNT4 | "UDP-GlcNAc:betaGal beta-1,3-N-acetylglucosaminyltransferase 4 [Source:HGNC Symbol;Acc:HGNC:15683]" | 9500 | -0.274 | -0.0272 | No |
| 108 | UGGT1 | UDP-glucose glycoprotein glucosyltransferase 1 [Source:HGNC Symbol;Acc:HGNC:15663] | 9516 | -0.275 | -0.0219 | No |
| 109 | PIGA | phosphatidylinositol glycan anchor biosynthesis class A [Source:HGNC Symbol;Acc:HGNC:8957] | 9571 | -0.280 | -0.0194 | No |
| 110 | A4GALT | "alpha 1,4-galactosyltransferase (P blood group) [Source:HGNC Symbol;Acc:HGNC:18149]" | 9766 | -0.297 | -0.0275 | No |
| 111 | CSGALNACT2 | chondroitin sulfate N-acetylgalactosaminyltransferase 2 [Source:HGNC Symbol;Acc:HGNC:24292] | 9773 | -0.298 | -0.0209 | No |
| 112 | POGLUT1 | protein O-glucosyltransferase 1 [Source:HGNC Symbol;Acc:HGNC:22954] | 9794 | -0.301 | -0.0153 | No |
| 113 | FUT3 | fucosyltransferase 3 (Lewis blood group) [Source:HGNC Symbol;Acc:HGNC:4014] | 9890 | -0.311 | -0.0153 | No |
| 114 | PLOD2 | "procollagen-lysine,2-oxoglutarate 5-dioxygenase 2 [Source:HGNC Symbol;Acc:HGNC:9082]" | 10086 | -0.330 | -0.0228 | No |
| 115 | EOGT | EGF domain specific O-linked N-acetylglucosamine transferase [Source:HGNC Symbol;Acc:HGNC:28526] | 10174 | -0.338 | -0.0215 | No |
| 116 | GBE1 | "1,4-alpha-glucan branching enzyme 1 [Source:HGNC Symbol;Acc:HGNC:4180]" | 10356 | -0.357 | -0.0272 | No |
| 117 | GALNT12 | polypeptide N-acetylgalactosaminyltransferase 12 [Source:HGNC Symbol;Acc:HGNC:19877] | 10414 | -0.362 | -0.0231 | No |
| 118 | B4GALT2 | "beta-1,4-galactosyltransferase 2 [Source:HGNC Symbol;Acc:HGNC:925]" | 10490 | -0.372 | -0.0201 | No |
| 119 | HAS2 | hyaluronan synthase 2 [Source:HGNC Symbol;Acc:HGNC:4819] | 10980 | -0.428 | -0.0482 | No |
| 120 | GALNT3 | polypeptide N-acetylgalactosaminyltransferase 3 [Source:HGNC Symbol;Acc:HGNC:4125] | 11062 | -0.440 | -0.0441 | No |
| 121 | FUT1 | fucosyltransferase 1 (H blood group) [Source:HGNC Symbol;Acc:HGNC:4012] | 11086 | -0.442 | -0.0353 | No |
| 122 | FUT2 | fucosyltransferase 2 [Source:HGNC Symbol;Acc:HGNC:4013] | 11459 | -0.497 | -0.0526 | No |
| 123 | B4GALT1 | "beta-1,4-galactosyltransferase 1 [Source:HGNC Symbol;Acc:HGNC:924]" | 11530 | -0.505 | -0.0461 | No |
| 124 | A4GNT | "alpha-1,4-N-acetylglucosaminyltransferase [Source:HGNC Symbol;Acc:HGNC:17968]" | 11568 | -0.510 | -0.0368 | No |
| 125 | CHSY1 | chondroitin sulfate synthase 1 [Source:HGNC Symbol;Acc:HGNC:17198] | 11628 | -0.521 | -0.0291 | No |
| 126 | POFUT2 | protein O-fucosyltransferase 2 [Source:HGNC Symbol;Acc:HGNC:14683] | 11725 | -0.537 | -0.0238 | No |
| 127 | B4GALT3 | "beta-1,4-galactosyltransferase 3 [Source:HGNC Symbol;Acc:HGNC:926]" | 11748 | -0.540 | -0.0126 | No |
| 128 | FUT6 | fucosyltransferase 6 [Source:HGNC Symbol;Acc:HGNC:4017] | 12067 | -0.612 | -0.0230 | No |
| 129 | UGT2B15 | UDP glucuronosyltransferase family 2 member B15 [Source:HGNC Symbol;Acc:HGNC:12546] | 12225 | -0.652 | -0.0198 | No |
| 130 | OGT | O-linked N-acetylglucosamine (GlcNAc) transferase [Source:HGNC Symbol;Acc:HGNC:8127] | 12440 | -0.721 | -0.0194 | No |
| 131 | EXTL3 | exostosin like glycosyltransferase 3 [Source:HGNC Symbol;Acc:HGNC:3518] | 12442 | -0.722 | -0.0022 | No |
| 132 | UGCG | UDP-glucose ceramide glucosyltransferase [Source:HGNC Symbol;Acc:HGNC:12524] | 12532 | -0.763 | 0.0090 | No |
| 133 | HAS1 | hyaluronan synthase 1 [Source:HGNC Symbol;Acc:HGNC:4818] | 12655 | -0.831 | 0.0192 | No |
Table: GSEA details [plain text format]

  

Fig 2: GOMF\_HEXOSYLTRANSFERASE\_ACTIVITY      
 Blue-Pink O' Gram in the Space of the Analyzed GeneSet

  

Fig 3: GOMF\_HEXOSYLTRANSFERASE\_ACTIVITY: Random ES distribution      
 Gene set null distribution of ES for **GOMF\_HEXOSYLTRANSFERASE\_ACTIVITY**
